# Differential Acetylation of Histone H3 at the Regulatory Region of *OsDREB1b* Promoter Facilitates Chromatin Remodelling and Transcription Activation during Cold Stress

**DOI:** 10.1371/journal.pone.0100343

**Published:** 2014-06-18

**Authors:** Dipan Roy, Amit Paul, Adrita Roy, Ritesh Ghosh, Payel Ganguly, Shubho Chaudhuri

**Affiliations:** 1 Division of Plant Biology, Bose Institute (Centenary Campus), P-1/12, C.I.T. Scheme VII M, Kolkta-700054, West Bengal, India; 2 School of Biotechnology, Yeungnam University, Gyeongsan, Korea; Duke University, United States of America

## Abstract

The rice ortholog of DREB1, *OsDREB1b*, is transcriptionally induced by cold stress and over-expression of *OsDREB1b* results in increase tolerance towards high salt and freezing stress. This spatio-temporal expression of *OsDREB1b* is preceded by the change in chromatin structure at the promoter and the upstream region for gene activation. The promoter and the upstream region of *OsDREB1b* genes appear to be arranged into a nucleosome array. Nucleosome mapping of ∼700bp upstream region of *OsDREB1b* shows two positioned nucleosomes between −610 to −258 and a weakly positioned nucleosome at the core promoter and the TSS. Upon cold stress, there is a significant change in the nucleosome arrangement at the upstream region with increase in DNaseI hypersensitivity or MNase digestion in the vicinity of *cis* elements and TATA box at the core promoter. ChIP assays shows hyper-acetylation of histone H3K9 throughout the locus whereas region specific increase was observed in H3K14ac and H3K27ac. Moreover, there is an enrichment of RNA PolII occupancy at the promoter region during transcription activation. There is no significant change in the H3 occupancy in *OsDREB1b* locus negating the possibility of nucleosome loss during cold stress. Interestingly, cold induced enhanced transcript level of *OsDREB1b* as well as histone H3 acetylation at the upstream region was found to diminish when stressed plants were returned to normal temperature. The result indicates absolute necessity of changes in chromatin conformation for the transcription up-regulation of *OsDREB1b* gene in response to cold stress. The combined results show the existence of closed chromatin conformation at the upstream and promoter region of *OsDREB1b* in the transcription “off” state. During cold stress, changes in region specific histone modification marks promote the alteration of chromatin structure to facilitate the binding of transcription machinery for proper gene expression.

## Introduction

The highly complex structure of chromatin imparts resistance to several nuclear processes including transcription [1], [2]. Nucleosome, the basic repeating unit of chromatin, is the site for the dynamic modifications which occur to generate ‘open’ or ‘closed’ chromatin configuration inside the cell. The N-terminal tails of histones are the major sites which undergo several covalent modifications including acetylation, methylation, phosphorylation, ubiquitination etc [3]. The combination of these modifications generates a “histone code” which promotes the accessibility and binding of nuclear factors to their cognate binding site during any nuclear processes by either directly weakening the electrostatic interactions between the histone octamer and the DNA wrapped around it or by acting as molecular signals facilitating the recruitment of chromatin remodelers or chromatin modifiers.

Plant being sessile organism has the ability to sense and respond quickly in response to environmental cues for its adaptation and survival. Abiotic stress such as drought, high salt content and temperature changes negatively influence both the growth and survival of plants [4]. Water stress imparted by drought and temperature severity is the most prevalent abiotic stress that limits plant growth and survival [5]. Plants respond and adapt to these conditions with an array of biochemical and physiological alterations. Multiple signalling events mostly governed by plant stress hormones like ABA, ethylene, JA or SA has been shown to regulate the stress responses of plants by modulating the expression of various stress responsive genes. It is becoming evident from recent studies that the expression of these stress responsive genes is regulated epigenetically through changes in histone modification or DNA methylation [6], [7]. Studies have shown that in response to drought stress there was region specific enrichment of H3K23ac and H3K27ac at the promoter and coding regions of the Arabidopsis drought responsive genes *RD29A*, *RD29B*, *RD20* and At2g20880 [8]. During hypoxia in rice seedlings, acetylation of histone H3 and the conversion of di-methyl H3K4 to tri-methyl H3K4 were observed in stress responsive genes *ADH1* and *PDC1* [9]. Dynamic changes in histone modifications has been reported in Tobacco BY2 and Arabidopsis cell lines where transient up-regulation of H3 phosphoacetylation (S10&K14) followed by histone H4 acetylation occur in response to high salinity, cold stress and ABA [10]. Interestingly, it was found for cold responsive genes *COR15a* and *AtGLOS3* that there was a decrease in H3K27me3 mark (transcription repression) for the transcription, in response to cold stress [11]. However, when these plants were brought back to normal temperature from cold stress, the transcription of *COR15a* and *AtGLOS3* decreased but the low H3K27me3 status was maintained in these plants for 3days at normal growth temperature. These findings suggest that rapid alteration of epigenetic marks and there reversibility with a potential to keep a memory to give plants the flexibility to respond to environmental stress.

The presence of a positioned nucleosome at the promoters prevents the binding of transcription factors to the promoter sites and hence inhibits transcription [12]. Therefore, during transcription activation, repositioning of nucleosome is required to increase accessibility of these promoters to transcription factors [13]. Studies have shown that there is a change in the nucleosome density at the promoter/enhancer region of certain stress responsive genes to establish an “open” conformation for the rapid binding of stress responsive transcription factor. ChIP analysis using antibody against c-terminal H3 indicate low nucleosome density under drought stress at the promoter region of *RD29A* and *RD29B* genes which contain DRE/CRT or ABRE elements [8]. However, there is no significant change in nucleosome occupancy at the coding region of these genes. Change in nucleosome occupancy was found to be dynamic in case of cold responsive genes *COR15a* and *AtGOL3* where again the H3 density decrease initially at the promoter region during transcription but the occupancy increases to normal within 1d after returning to normal temperature [11]. These nucleosome-free regions generated at the promoter and *cis*-regulatory element during transcription activation showed enhanced DNaseI hypersensitive sites (DHS) in many plant genes such as Arabidopsis *ADH* [14], tomato proteinase inhibitor I [15] and *Chlamydomonas HSP70A* and *RbcS2* [16]. Genome-wide high resolution mapping of DH sites in Arabidopsis [17] and rice genome [18] indicate that most of the region near the DHS is nucleosome void and are associated with RNA Polymerase II binding site. Interestingly, the region associated with DH sites especially promoters or transcription start site, showed an overall decrease in histone modifications compared with immediate adjacent region, again indicating that these regions might be of nucleosome depleted/dynamic region. Also, the DNA sequences contained within the DH sites were found to be hypomethylated, consistent with transcription activation [18]. Thus, the epigenetic modifications govern the overall chromatin structure of a particular region during transcription and decides the DNase I hypersensitive sites of that region.

The Dehydration responsive element binding proteins (DREBs) especially *DREB1a*, *DREB1b*, *DREB2a* and *DREB2b* are the major transcription factors that regulate cold, high salt or dehydration inducible gene expression in plants [19], [20], [21]. These proteins specifically bind to DRE/CRT elements and activate the transcription of stress responsive genes [22], [23]. Extensive research with a wide variety of plants has shown that DREB proteins were differentially expressed in response to different abiotic stresses. Expression of Arabidopsis as well as rice *DREB1/CBF* genes has been shown to be induced by cold stress but not by dehydration or salt stress [20], [21]. Interestingly, transgenic rice over-expressing *OsDREB1* genes not only show heightened tolerance to freezing temperature but also to drought or high-salt condition [24]. In this study, we have investigated the epigenetic changes involved during the expression of *OsDREB1b* genes in rice during cold stress. Our results indicate that hyperacetylation at the promoter and upstream region of *DREB1b* gene promotes the alteration in the chromatin structure during transcription. These changes in the chromatin structure are not random but occur in a region specific manner to increase the accessibility of stress responsive TF binding sites for transcription.

## Materials and Methods

### Plant Material, Growth Conditions and Stress Treatment

*Oryza sativa* L. cv. IR-64 seeds were germinated according to protocol described elsewhere [25] Seeds of *Oryza sativa* L. cv. IR-64 were surface sterilized with 0.1% (w/v) HgCl_2_ for 15 min, washed and allowed to germinate over water-soaked sterile gauge in trays at 37°C in dark for 3 days. The germinated seedlings were grown in water-soaked sterile gauge in trays in presence of 0.25X Murashige and Skoog (MS) complete media at 30°C under 16 h light and 8 h dark photoperiodic cycle with 50% relative humidity and 700 lmol photons m^−2^s^−1^ for the desired period in a plant growth chamber. For cold-stress treatment, the 17 day-old seedlings were transferred to 4°C for varying time periods ranging from 2 hrs to 16 hrs; while the control plants were maintained at 30°C. For recovery experiments, 17-cold seedlings were first incubated at 4°C for 2 hrs and then transferred to growth chamber at 30°C for indicated time period.

### RNA Isolation and Northern Blot Analysis

RNA samples were isolated 17 days old rice seedlings using Trizol reagent (invitrogen) as described by manufacturer protocol. 12 µg of total RNA from control and stress treated samples were used for RNA blot and hybridised with probes specific for the 3′ region of *OsDREB1b (+648 to +843)* and *OsDREB2a (+3411 to +3600).* Rice actin (OSJNBa0005K07) gene was used as internal control. The primers used to amplify the 3′region of *OsDREB1b*, *OsDREB2a* and actin were listed in Table S1.

### Nuclei Extraction

Rice seedlings (10–12 grams) were homogenized using liquid Nitrogen in 200 ml ice cold extraction buffer1 (0.4M Sucrose; 10 mMTris-HCl, pH 8.0; 10 mM MgCl_2_; 5 mM β-mercaptoethanol; 10 mM spermidine; 1 mM PMSF and Protease cocktail inhibitors). The extract was filtered twice through two layers of Miracloth and the filtrate was centrifuged at 4000 rpm for 30 minutes at 4°C. The pellet was resuspended gently with 2 ml of extraction buffer 2 (0.25M Sucrose; 10 mMTris-HCl, pH 8.0; 10 mM MgCl_2_; 1% Triton X-100; 5 mM β-mercaptoethanol; 10 mM spermidine; 1 mM PMSF and Protease cocktail inhibitors) and centrifuged at 13000 rpm at 4°C for 10 minutes. The pellet was re-suspended again in 0.5 ml of extraction buffer 2 and the solution was layered slowly on top of 0.5 ml of extraction buffer 3(1.7M Sucrose; 10 mMTris-HCl, pH 8.0; 2 mM MgCl_2_; 0.15% Triton X-100; 5 mM β-mercaptoethanol; 10 mM spermidine; 1 mM PMSF and Protease cocktail inhibitors ) and centrifuged at 13000 rpm at 4°C for one hour. The nuclear pellet was washed consecutively in washing buffer (50 mM Tris-HCl, pH8.0; 5 mM MgCl_2_; 10 mM β-Mercaptoethanol; 20% Glycerol, 0.25%Triton-X 100) and storage buffer (50 mM Tris-HCl, pH8.0; 5 mM MgCl_2_, 25% glycerol and 10 mM β-Mercaptoethanol) and was finally resuspended in storage buffer for subsequent experiments.

### Micrococcal Nuclease and DNaseI digestion of nuclei

Nuclei resuspended in storage buffer were resuspended in Storage Buffer supplemented with 1.5 mM CaCl_2_ and incubated with increasing concentration (as indicated in figure legend) of MNase (Worthington)_._ The reaction mixture was incubated at 37°C for 20 minutes and was then terminated with 1%SDS and 50 mM EDTA. The nucleosomal DNA was extracted with equal volume phenol:chloroform (v/v). For DNaseI digestion, the nuclei were resuspended in DNaseI buffer (25 mM Tris-HCl, pH 8.0; 10 mM MgCl_2_; 50 mM NaCl; 10% glycerol; 0.2 mM DTT) and digested as indicated in figure legend. For Indirect end-labelling experiments, the MNase digested chromatin was further digested with restricted endonuclease as indicated and the purified DNA was separated in a 1% agarose gel, transferred on nylon N+ membrane and Southern hybridized by standard protocols [26] using radio-labelled probes corresponding to different region of *OsDREB1b* locus.

### Chromatin Immunoprecipitation

ChIP assay was adapted from protocols described previously [27], [28]. Briefly, chromatin solution was pre-cleaned by incubating the crude extract with Protein A or G beads before using it for immuprecipitation assay. 600 µl of sonicated chromatin solution was combined with following antibodies: 6 µl of H3 unmodified (ab1791, abcam), 6 µl of H3K4me3 (ab8580, abcam), 6 µl of H3K9ac (ab0812, abcam), 6 µl of H3K14ac (ab46984, abcam), 6 µl of H3K27ac (ab4729, abcam) and 6 µg of H3K27me3 (ab6002, abcam), anti RNA Pol II (8wg16; ab817, abcam). The solutions were then incubated overnight at 4°C on a rotation wheel. The immunocomplexes were isolated by 40 µl of protein A/G–Sepharose beads (50% slurry pre-absorbed with 0.1% BSA and 100 µg/ml of salmon sperm DNA). The beads were washed once with low salt wash buffer (150 mM NaCl, 0.1% SDS, 1% Triton X-100, 2 mM EDTA, 20 mM Tris- HCl, pH 8.0); once with high salt wash buffer(500 mM NaCl, 0.1% SDS, 1% Triton X-100, 2 mM EDTA, 20 mM Tris- HCl, pH 8.0), once with LiCl wash Buffer(0.25% LiCl, 1% Nonidet P-40, 1% sodium deoxycholate, 1 mM EDTA, 10 mM Tris-HCl, pH8.0) and twice with TE. The immunoprecipitated chromatin was eluted with elution buffer containing 250 µl of 1% SDS and 0.1 M NaHCO_3_ at 65°C for 15 min, mixed with 20 µl of 5M NaCl and incubated overnight at 65°C to reverse the formaldehyde cross-linkages. The next day, 10 µl of 0.5M EDTA; 20 µl of Tris-HCl, pH 6.5 and 1 µl of Proteinase K (20 mg/ml) was added to each elute and incubated at 45°C for 1 hr and extracted with phenol: chloroform: isoamyl alcohol (25∶24:1) and chloroform. After precipitation with ethanol in the presence of 50 µg of glycogen, the purified DNA pellets were suspended in 50 µL of TE and analyzed by Q-PCR.

For Western blot, nuclear protein was isolated by lysing the nuclei isolated from control and stress treated plants. The nuclear extract was run on 15% SDS-PAGE, blotted on PVDF membrane and probed with antibodies against histone H3, H3K9ac, H3K14ac and H3K27ac.

### PCR Conditions and Data Analysis

Real time PCR was carried out in 20 µl reaction volume containing 1X buffer with Syber Green and 0.25 µM primers (DyNAmo ColorFlash SYBER green, Thermo scientific). For each set, input samples were diluted and used for generating a standard curve. The amount of DNA pulled down for individual region was calculated using input standard curve. PCR was carried out with primers specific for the promoter and upstream region of *OsDREB1b* and *OsDREB2a*. The % input values thus obtained for these regions were calculated from the standard curve. To normalise the ChIP data, the rice actin promoter region was used. The % input values for DREB locus were normalised with the values obtained for actin promoter and represented as “normalised % input” as done by Pavangadkar et al [29]. The error bar in each graph indicate standard error (SE) generated from at least three independent ChIP experiments for control and stress treated samples. For regions of *OsDREB1b* ChIP results were analysed by semi-quantitative PCR. The PCR was done with 1 µl diluted ChIP DNA (1∶2.5 times) in a 30 µl reaction volume containing 0.15 mM primers, 1.5 mM MgCl_2_, 0.15 mM dNTPs and 5% DMSO. The data was also normalised with values obtained for actin promoter obtained under similar PCR condition. To determine the significance of the results, the values obtained for stress treated samples were compared with control and analysed by student’s *t* test. The significant changes (P≤0.05) were marked in the figures. The primers used for ChIP analysis were listed in Table S1.

### Enzyme Accessibility Assay

Enzyme (MNase and DNase I) accessibility assay to monitor chromatin remodelling in control and stressed nuclei was done as described by Chua et al. (2001). Nuclei from control and stress treated seedlings were digested with MNase (30 U/ml) and DNase I (5 U/ml) for different time period (as indicated in the figure legend). The digested mixture was extracted with equal volume of Phenol: Chloroform (1∶1 v/v) and precipitated with ethanol. Three replicates of each nuclease treatment were performed and analysed by quantitative PCR. The enzyme accessibility at the actin promoter and *OsDREB2a* was used as control locus in this case. The amount of PCR product obtained for different digestion time point was normalised with the amount present at 0 time point. The normalised signal was plotted against time to compare the degradation rate for each condition.

## Results

### Induction of OsDREB1b Transcript in Response to Cold Stress

**Figure 1:**
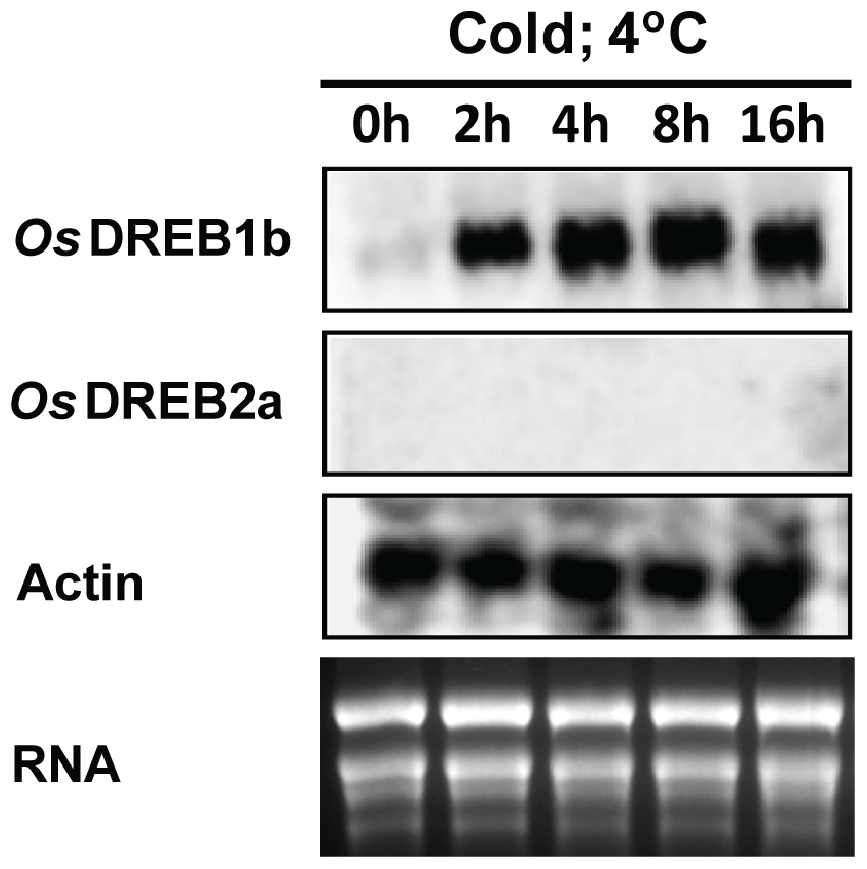
Expression profile of *OsDREB1b* and *OsDREB2a* gene under cold and high salt stress condition. The transcript of *OsDREB1b* and *OsDREB2a* gene was monitored by northern blot analysis using gene specific probes generated from 3′ end of the gene. The rice actin gene (OSJNBa0005K07) was used as internal control.

The expression of *OsDREB1b* genes during cold stress was analysed using northern blot hybridization. For this, RNA was isolated from 17 days old rice seedlings (*Oryza sativa Indica, var IR 64*) treated at cold temperature (4°C) for different time periods and the blot was hybridised with probes generated from the 3′ terminal of DREB genes (Figure 1). The result indicates accumulation of *OsDREB1b* transcripts from 2 hrs of cold treatment and maintained at higher level almost 16 hrs of cold treatment compared to control plant. There was no significant induction of *OsDREB2a* gene, which encodes another DRE binding protein, in response to cold stress. The expression data was consistent with previously published data [21] and gave us the scope to analyse the chromatin structure of the *OsDREB1b* loci at two different transcriptional states: an activated state (Cold, 4°C, 2 hrs and 4 hrs) and a basal state (Control).

### Nucleosome Organization at the Promoter and Upstream Region of the *OsDREB1b* Gene

**Figure 2:**
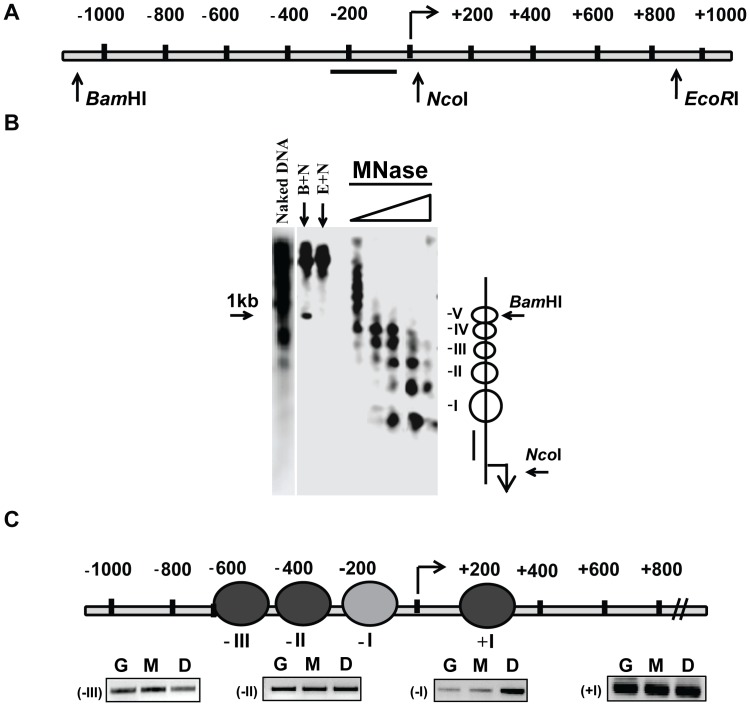
Mapping of nucleosome positions at the promoter and upstream region of *OsDREB1b* locus. (A). Chromatin isolated from 2–3 weeks old rice seedlings were partially digested with increasing concentration of MNase, and probed with DNA from the core promoter region (−74 to −232) (B) Determination of the nucleosome positions at the promoter and upstream region *OsDREB1b* locus by PCR based approach. The positions of the nucleosomes are given relative to the *OsDREB1b* locus. G denotes genomic DNA, M denotes mono-nucleosomal DNA and D denotes di-nucleosomal DNA. Black shaded ovals denote a well positioned nucleosome and a grey shaded oval represents a partially positioned nucleosome.

In order to understand how the nucleosomes alter during the cold induced transcription of *OsDREB1b* gene, we initially mapped the nucleosome arrangement of promoter proximal region of *OsDREB1b* locus by MNase digestion combined with indirect end-labelling technique [30]. In this case, naked DNA and nuclei were first digested with increasing concentration of MNase followed by *Nco*I restriction enzyme digestion and hybridised with DNA corresponding to the promoter region (−74 to −232) as shown in figure 2A. The MNase digestion profile indicate that there are approximately five nucleosomes within 1kb region (−74 to −1118) upstream of the putative transcription start site (Figure 2A, ii). The result further indicates that under transcriptionally inactive state, the linker region between nucleosome −II/−III is less accessible to enzyme, as the cleavage occurs only at higher enzyme concentration. This phenomenon can be due to the presence of small linker region between nucleosomes −II/−III leading to reduced accessibility of the enzyme. The linker DNA connecting nucleosome −III/−IV and −IV/−V on the other hand seems to be more accessible to MNase with increasing concentration of the enzyme.

We next determined the position of these nucleosomes at 30–50 bp resolution at the upstream regulatory region of *OsDREB1b* using PCR based approach (see method). In this approach, the region of interest was divided by a series of primers that were15–25 bp apart from each other so that a combination of each primer set (forward and reverse primer) can amplify 150 to 200 bp DNA fragment that may correspond to at least one nucleosome (Figure S1). Nuclei were again digested with increasing concentration of MNase, mono- and di-nucleosomal DNA fraction along with genomic DNA were then used as PCR templates. The primer set that generate PCR signal intensity from mononucleosomal DNA template comparable to the intensity derived from both di- nucleosomal and genomic DNA template has been considered to cover a region that has a well positioned nucleosome. If the amplification for a particular region occurs only from di-nucleosomal DNA fraction then either the region has two nucleosomes in tandem or there is a partially positioned or not well positioned nucleosome at that region. With this logic we have determined the position of three nucleosomes at the upstream region of *OsDREB1b*: nucleosome −I(−40 to −232), nucleosome −II(−258 to −415) and nucleosome −III (−440 to −610) (Figure 2B and Figure S1). Nucleosomes at positions −258 to −415 (−II) and −440 to −610 (−III) seems to be well positioned as the amplification from mononucleosomal fraction is comparable to both genomic and di nucleosomal DNA template. However, nucleosome at position −40 to −232 seems to be partly/loosely positioned as the amplification in this region is mostly from di-nucleosomal DNA and less from mono fraction. It is also possible that the region may have two tandem nucleosomes which are less accessible to MNase. Since, the indirect end-labelling result clearly indicate a mono nucleosomal band corresponding to −I position; the possibility of tandem nucleosome does not exist for this region. Rather the result suggests that the region −40 to −232 is occupied by a less strongly positioned nucleosome. We have also mapped one positioned nucleosome at +157 to +307 which is 200 bp downstream from transcripstion start site.

### Hyper-acetylation of Histone H3 Promotes Induction of OsDREB1b Gene during Cold Stress

Higher order chromatin structure imparts structural constraint for the transcriptional machinery to move. Changes in the post-translational modifications of histone N-terminal tail residues particularly acetylation of histone residue, play a pivotal role in determining the chromatin state of a gene and controlling transcription. In order to understand how histone acetylation changes in *OsDREB1b* locus during transcription activation in response to cold stress, we monitored the acetylation status of H3K9, H3K14 and H3K27 residues respectively, using ChIP assay. The promoter proximal region and TSS of *OsDREB1b* locus (−800 to +350 bp) was divided into 4 different regions: region Ia (−232 to −40); region Ib: (−415 to −258); region II (−610 to −440) and region III (+157 to 307). The primers were designed against all the four regions and used for amplifying the corresponding fragment from immunoprecipitated chromatin. The immuprecipitated DNA for region Ib and II of *OsDREB1b* was analysed by real time PCR (Syber green based). For the region Ia (−232 to −40) and region III (+157 to 307) we were unable to quantitate ChIP data using Real time PCR. The exact reason for this failure is not known. The only possibility is that the promoter and TSS region from −230 to +250 of *OsDREB1b* has high GC content (>75%) and previous reports have suggested that Syber green dye prefer AT rich dsDNA compared to GC rich [31]. This may have led to poor amplification plot (delayed Ct values) in real time PCR. Since these two regions were very important for the regulation of *OsDREB1b* gene we proceeded our analysis with our semi-quant data. We have used actin promoter region as internal control to normalise the ChIP data for real time as well as semi-quantitative data.

#### Promoter and upstream region of *OsDREB1b* gene

**Figure 3:**
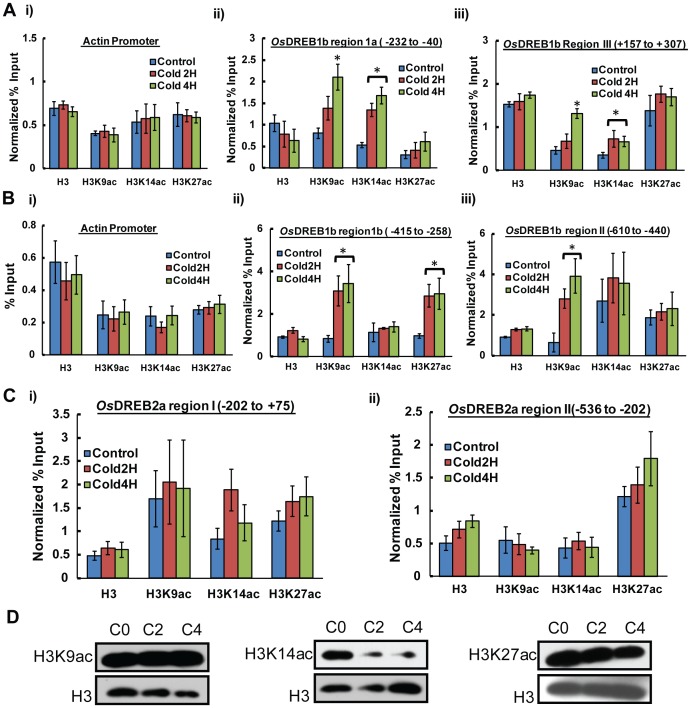
Alteration of histone H3 modifications during cold stress. Relative change in Histone H3 acetylation (H3K9ac, H3K14ac, and H3K27ac) during cold stress at (A) region Ia (−232 to −40) and region III (+157 to +307) (B) region Ib (−415 to −246) and region II (−610 to −440) of *OsDREB1b* gene. (C) Relative change in histone modifications at promoter and upstream region of *OsDREB2a* during cold stress. Samples were analysed by real time PCR except (A). The mean values for each region were normalised to Actin promoter values. Error bar represent standard error (SE) where number of independent experiments (n) = 3. The significance of the results were analysed by student’s *t* test and the significant changes (P≤0.05) were marked by *. (C) Western blot showing H3K9ac, H3K14ac and H3K27ac signal in whole cell extract isolated from control and cold stress treated rice seedlings.

ChIP analysis shown in Figure 3 and Figure S2 indicate that there is an increase in histone H3 acetylation marks at the promoter and upstream regulatory region (region Ia, Ib and II) of *OsDREB1b* gene during cold stress. Histone H3K9 acetylation shows 2 to 4 fold (p<0.05) increases throughout the 800 bp upstream region of *OsDREB1b*. Along with H3K9 acetylation there are other H3 residues which get differentially enriched in a region specific manner in response to cold stress. Region Ia which covers the predicted TATA box and TFIIb binding site shows an significant enrichment in H3K14 acetylation mark (∼3 fold, p<0.02) upon transcription activation (Figure 3A, ii) whereas region Ib, which is upstream to region 1a, shows enrichment of H3K27ac (∼3 fold, p<0.03) marks in response to cold treatment (Figure 3B, ii). Region II on the other hand has no significant change in the acetylation of H3K14 or H3K27 other than unique enrichment of H3K9ac (4 to 6 fold, p<0.03) during cold stress (Figure 3B, iii). The H3 occupancy in these regions does not change significantly during cold stress (Figure 3A, i and 3B, i), negating the possibility of nucleosome lose in these region during cold stress.

#### Coding region of *OsDREB1b* gene

The coding region of *OsDREB1b* gene shows at least 2-fold (p<0.03) increase in H3K14 acetylation marks during cold stress along with gradual increase in H3K9 acetylation in cold treated nuclei (4 hr stress time point) compared to control (Figure 3A, iii). However, there was no significant change in histone modification marks in the region downstream from transcription termination site of *OsDREB1b* (data not shown). To further investigate whether these changes in histone marks observed in the *OsDREB1b* locus is because of a global change of post translational modifications of histone H3 in response to cold, we performed western blot with total protein extract isolated from control and cold stress treated plants. Our results indicate that there is no significant change in H3K9 and H3K27 acetylation at the global level in response to cold stress whereas H3K14ac gets reduced after cold treatment (Figure 3D). Therefore, the changes observed in H3K9, K14 and K27 acetylation status in *OsDREB1b* locus is region specific and not due to a global change in the chromatin in response to cold stress.

*OsDREB2a* encodes another member of DRE binding transcription factor family which is transcriptionally repressed in response to cold stress but induced by salt or drought stress. We have also examined the changes in histone modifications at the promoter proximal region of *OsDREB2a* locus in response to cold stress. The results indicate that there is no significant fold change in acetylation marks of H3K9, K14 and K27 residue in response to cold stress within 800 bp region which span the upstream region and promoter region of *OsDREB2a* (Figure 3C). Collectively these ChIP study with histone acetylation specific antibodies indicate that the increase in acetylation of H3 residues at the promoter and upstream region of *OsDREB1b* is highly locus specific and is correlated with its transcriptional activation during cold stress.

### RNA Pol II Occupancy at *OsDREB1b* Locus during Cold Stress

**Figure 4:**
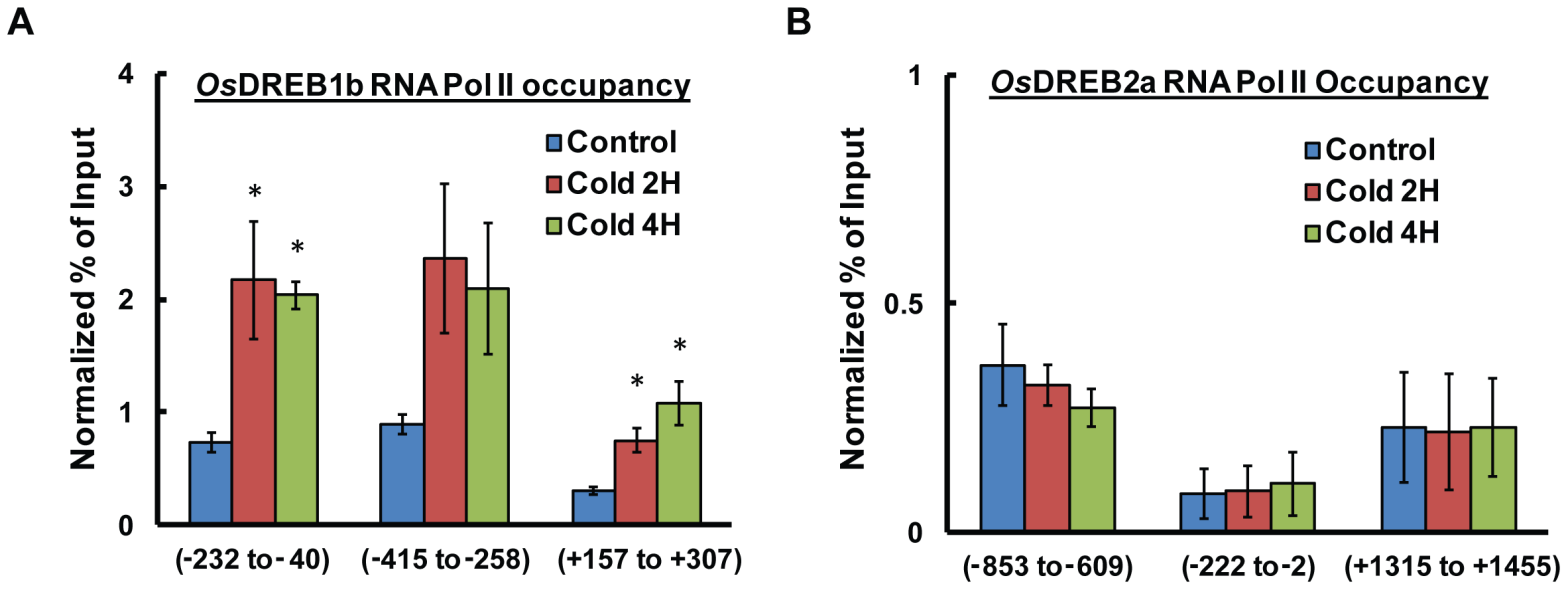
Change in RNA polymerase and nucleosome occupancy during cold stress. The relative enrichment of RNA Pol II binding at the promoter and coding region of (A) *OsDREB1b* and (B) *OsDREB2a* was determined by ChIP assay using antibody against RNA Pol II CTD (8WG16). The ChIP data was normalised to Actin promoter values. Error bar represent standard error (SE) where number of independent experiments (n) = 3. The significance of the results were analysed by student’s *t* test and the significant changes (P≤0.05) were marked by *.

To determine whether the above changes in the histone H3 marks at the *OsDREB1b* locus promote the accessibility of RNA Pol II at the promoter region, ChIP experiment using antibody against the initiating RNA polymerase II (8WG16; Abcam), was carried out. The ChIP results clearly indicate enrichment of initiating RNA Pol II predominantly in region Ia of *OsDREB1b* promoter in response to 2 hr and 4 hr of cold stress (2 fold, p≤0.03) (Figure 4A). As mentioned earlier, region Ia represent the genomic region upstream of transcription start site and has the predicted TATA box element and TFIIb binding site. The RNA Pol II ChIP result is consistent with our northern blot analysis where the *OsDREB1b* transcript was seem to increase in response to cold stress 2 hr onwards. Interestingly, there was also a 3 fold change (*p*<0.001) in increase in RNA Pol II occupancy around TSS during cold stress. In case of transcriptionally repressed *OsDREB2a* locus, no significant change in RNA Pol II occupancy was observed during cold stress (Figure 4B). The result is in consonance with transcript data showing no expression of *OsDREB2a* in response to cold stress.

### Alteration of Chromatin Structure at the Promoter Region of DREB1b Loci upon Transcription Activation

The positioning of nucleosomes on promoters and upstream regulatory elements is inhibitory to transcription as it occludes the access of both transcription factors and the transcriptional machinery to their cognate sites. Transcriptional activation therefore requires active repositioning of nucleosomes which can in part be accomplished by changes in the epigenetic marks that facilitates “opening” of the chromatin structure at a locus [12]. Alteration of chromatin structure at the promoter and upstream region of *OsDREB1b* locus during transcriptional activation was examined by measuring the changes in nuclease accessibility (MNase and DNaseI). Both MNase and DNaseI have different DNA digestion characteristics and exhibit different sequence specificities. In chromatin context the digestion profile of these enzymes is influenced by not only the presence of nucleosomes but also the conformation assumed by nucleosomes at different transcriptional states. Hence, the changes in the accessibility of these two endonucleases at the *OsDREB1b* locus at different stages of cold stress should reflect the conformational changes in the chromatin structure in response to the environmental cue.

Nuclei representative of all three transcription states (control, cold 2 h and 4 h) were isolated and digested with MNase and DNase I for increasing time points. DNA was isolated; subjected to quantitative PCR reaction using primers for different region of *OsDREB1b* locus and normalised to the amount of DNA present at “0” digestion time point. The normalised values where then plotted against time to compare degradation rates between three transcription states.

**Figure 5:**
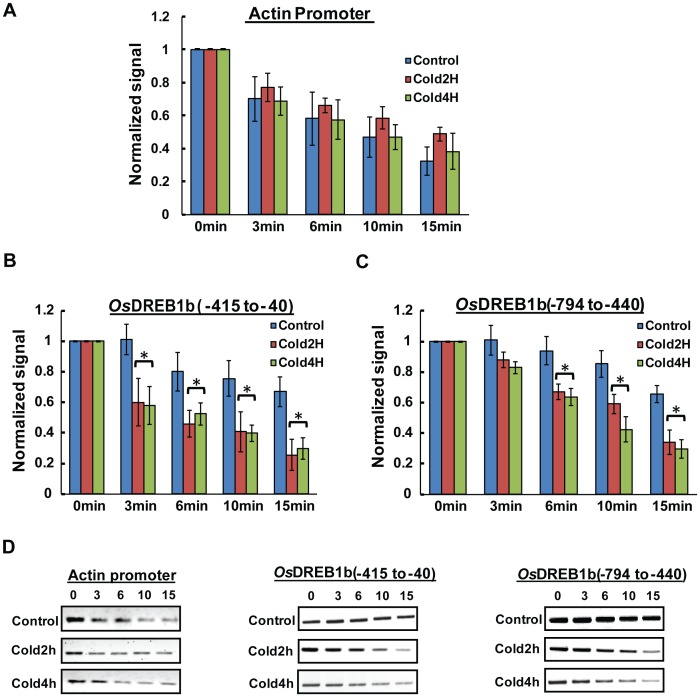
Change in chromatin structure at the promoter and upstream region of *OsDREB1b loci* using micrococcal nuclease digestion. MNase accessibility in control and cold stress treated nuclei (2 Hr and 4 Hr) was detected with quantitative PCR based method. Nuclei were digested with MNase (30 U/ml) for increasing time period as indicated. The isolated DNA was used for PCR reaction with primers specific for promoter and upstream region. The amount of DNA amplified at each time point was normalised to that at time 0 and plotted against time to compare the rate of degradation. The data represented here is a mean of three independent experiments with standard error bars and statistically significant values were marked with *. (A and D) rate of degradation for actin promoter; (B, C, D and E) *OsDREB1b locus*.

**Figure 6:**
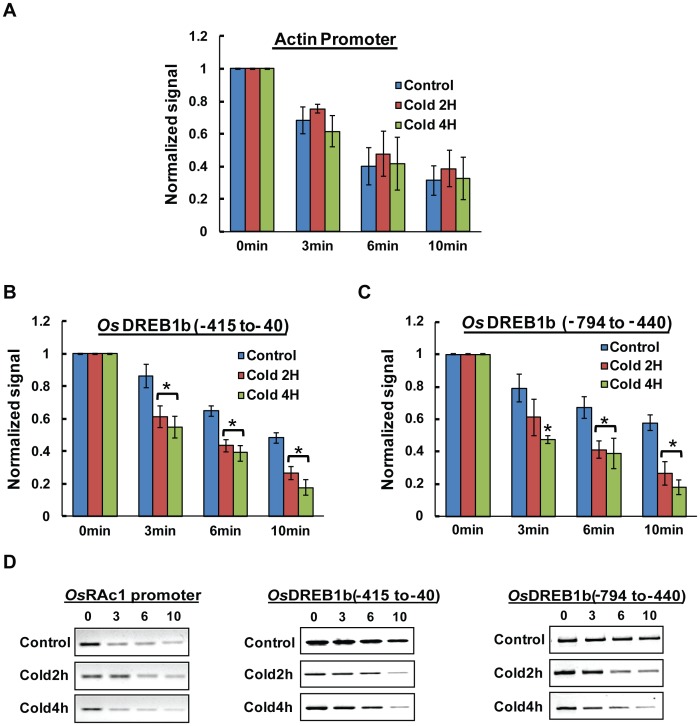
Change in DNase I accessibility at the promoter and upstream region of *OsDREB1b loci*. Relative DNase I accessibility in control and cold stress treated nuclei (2 Hr and 4 Hr) was detected with PCR based method. Nuclei were digested with DNase I (5 U/ml) for increasing time period (0,3,6,10 min). The isolated DNA was used for PCR reaction with primers specific for promoter and upstream region. The amount of DNA amplified at each time point was normalised to that at time 0 and plotted against time to compare the rate of degradation. The relative rate of accessibility for actin promoter (A and D) and *OsDREB1b* (B, C, D and E) and The data represented here is a mean of three independent experiments with standard error bars. Statistically significant values were marked with *.

Nuclease accessibility experiment shows, the rate of degradation of the amplification at region I (Promoter proximal:−415 to −40) and II (Upstream:−794 to −440) of *OsDREB1b* locus in cold treated samples were enhanced compared to control nuclei. Based on three independent experiment result it was found that in cold treated nuclei, 50% decrease in MNase digestion was observed within 10 min of incubation in both region I (p<0.02) (Figure 5B and D) and region II (p<0.04) (Figure 5C and D) and within 6 min of incubation for DNaseI digestion [Figure 6B and D for region I (p<0.01); Figure 6C and D for region II (p<0.03)]. For control plants, degradation was observed especially for DNaseI digestion but the rate is much slower compared to cold treated nuclei. These results clearly indicate that chromatin structure of 800 bp upstream region of *OsDREB1b* attains an open structure during cold exposure compared to plants that were grown under normal temperature. We have also monitored the chromatin structure of transcriptionally active Actin and transcriptionally repressed *OsDREB2a* gene in cold treated nuclei. The promoter region of Actin shows degradation both in control and cold treated samples and the rate in these nuclei were found to be comparable (Figure 5 A and D for MNase and Figure 6A and D for DNaseI). For transcriptionally repressed *OsDREB2a* gene, the rate of degradation both in control and stress treated nuclei were relatively slow and almost similar for the promoter and upstream region (Figure S3). Increase in Micrococcal nuclease and DNase I accessibility at the promoter proximal region during transcription reflects alteration in chromatin structure. This change in accessibility of endonuclease has been demonstrated during the transcription of many genes including pea plastocyanin gene (*PetE*) [28] or *nfc 102* gene of maize during UV-B treatment [32]. The enhancer/promoter region of these genes attains a closed chromatin conformation during transcriptionally inactive state. During the transcription activation, there is an increase in the accessibility of MNase and DNaseI indicating that the region attained open chromatin structure. Interestingly, the transcription of these genes is also associated with increase in histone H3 and H4 acetylation at the promoter region as observed in *OsDREB1b* gene in our case.

### Transcription Repression of *OsDREB1b* upon Return to Normal Temperature

**Figure 7:**
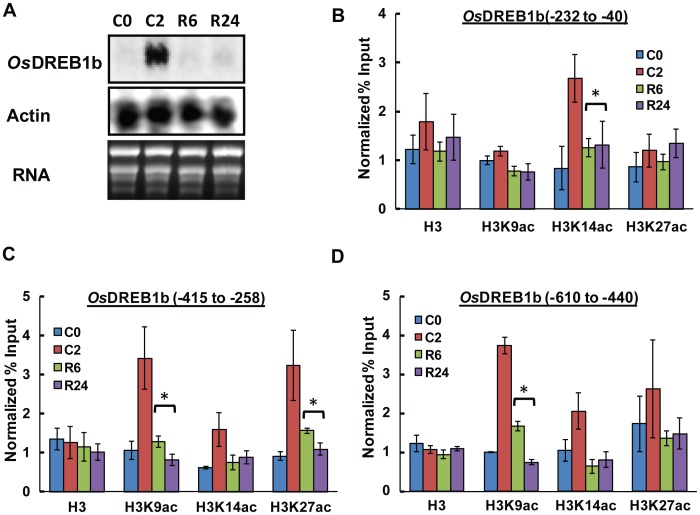
Relative fate of *OsDREb1b* transcription and histone modifications during cold exposure and subsequent return to normal growth temperature. 17 days old rice seedlings were subjected to cold stress and then recovered to normal growth temperature for 6(A) Comparison of *OsDREB1b* transcript level when plants were returned to normal temperature after cold stress. (B, C and D) Comparison of histone modifications at the *OsDREB1b* locus during recovery after cold stress. The data represented here is a mean of three independent experiments with standard error bars. Statistically significant values were marked with *.

We were also interested to see the effect on *OsDREB1b* transcription when cold-exposed plants were returned to a normal growth temperature. 17 days old rice seedlings were exposed to cold temperature (4°C) for 2 hrs and then allowed to grow at normal temperature for 6 hrs and 24 hrs. Northern analysis indicates that the level of *OsDREB1b* which was highly elevated during cold stress, reaches basal level within 6 hrs of incubation at normal growth temperature (Figure 7A). Thus, it appears that there is complete repression of cold induced transcription of *OsDREB1*b within 6 hrs of incubation at normal temperature.

We next investigate the fate of histone modifications at the promoter region of *OsDREb1b* locus when these stress treated plants where returned to normal temperature. The results indicate that the region specific increase of histone acetylation at H3 K9, K14 and K27 residue in the *OsDREB1b* locus recovered to initial level like control plants in a similar way as transcript level (Figure 7B, C and D). There is no change in histone H3 occupancy at the promoter region during recovery state. These results together indicate that the acetylation of specific residues of Histone H3 is directly correlated with the increase in the *OsDREB1b* transcription and these two processes are not independent events.

## Discussion

### Nucleosome Architecture of *OsDREB1b* Promoter and Upstream Region

In order to better understand the transcription regulation of rice *OsDREB1b* gene during cold stress, we determined the chromatin structure of 1 kb upstream region of *OsDREB1b* locus. Low resolution nucleosome map shows the presence of five nucleosomes within this region (Figure 2A). PCR based nucleosome occupancy result indicates that nucleosomes −II and −III are well positioned with a short linker region. The nucleosome −I which is mapped between −232 to −40, is weakly positioned and hypersensitive to MNase digestion. The result also suggests that the first 150 bp region downstream of TSS is nucleosome void region (Figure 2B). Interestingly, first 200 bp region downstream of TSS of *OsDREB1b* is highly GC rich (>75%) and studies have shown that high GC content sequence does not favour nucleosome occupancy and promote nucleosome depleted region [33], [34]. A previous study on mammalian promoters point out that the promoter region with high GC content (55%–93%) have less nucleosome density around +I and resulting in an increase of nucleosome depleted region resulting in the shift of nucleosome position from +I to the +II and +III [35]. Interestingly, *OsDREB1b* nucleosome arrangement indicates the presence of a nucleosome within +157 to +307 leaving a 200 bp weakly positioned sequence around TSS for nucleosome occupancy. Furthermore, our nucleosome mapping analysis was found to be in consonance with the Kaplan distribution model [36]. Taken together, the salient features of the nucleosome architecture at the promoter region of *OsDREB1b* are: (i) weakly positioned −I and +I nucleosome providing heterogeneous population of nucleosome occupancy at the core promoter and TSS during transcriptionally repressed state. The nature of this nucleosome occupancy introduces competition for forming a stable pre-initiating complex for initiating transcription (ii) the two positioned nucleosomes flanking the TATA box and TSS provides partial blockage for the progression of RNA polymerase during transcriptionally repressed state.

#### Changes in nucleosome structure in the regulatory region during transcription activation

Various studies in yeast and animal system have shown that the disruption of nucleosome structure at the regulatory regions is pre-requisite for the onset of transcription [13]. Alteration of chromatin structure during transcription shows increase in DNase I hypersensitive sites (HS) at the promoter and upstream region of a gene, indicating the open chromatin structure to expose the *cis*-element for transcription factor binding. Yeast *PHO5* transcription is a well studied model showing increase in DNase I HSs at the upstream region of the locus for the binding of transcription factor Pho4 [37], [38]. Changes in chromatin structure has been well studied for many plant genes like Arabidopsis *Adh* and *HSP18*, tomato proteinase inhibitor-1, Chlamydomonas *HSP70A* and *RbcS2* gene during transcription activation. In each case, the DNase HSs were found to be extended in the regulatory region like promoter, enhancer and other *cis* elements during transcription “on” state.

Closed chromatin conformation at the promoter and upstream region suppress the transcription of *OsDREB1b* gene in control plants as evident by transcription result (Figure 1). During transcription “on” state there is an increase in endonuclease accessibility in the promoter (region I) and upstream region (region II) resulting in a more open conformation. This open chromatin structure promotes transcription rate which increases in cold treated plants. Our results indicate that the chromatin conformation of 800 bp upstream region of *OsDREB1b* regulates of its transcription in response to external stimuli (Figure 5 and 6). Interestingly, transgenic Arabidopsis expressing GUS gene under the 800 bp promoter region of rice *OsDREB1b* gene showed GUS expression in response to variety of stresses indicating that the region contain regulatory cis- elements for stress-induced expression [39].

Sequence analysis and sequence prediction software shows putative TATA box and TFIIB binding sites reside within the first 100 bp sequence upstream from TSS of *OsDREB1b* gene. There are many stress responsive elements like ABRE and DRE core elements present within the region of nucleosome −I (−232 to −40). Many stress responsive TF binding sites with high score; mainly ABRE elements, bZIP binding sites, Myc like HLH binding sites are found to be masked within nucleosome −II and nucleosome −III of *OsDREB1b* gene. Hence, in order to obtain proper threshold of transcript of *OsDREB1b* during cold stress, nucleosomes remodeling seems to be a pre-requisite at promoter proximal region to uncover these *cis* elements.

### Hyperacetylation of Upstream Nucleosomes is Pre-requisite for the Alteration of Chromatin Structure

Yeast and mammalian studies have shown that HATs are an important component of transcription co-activator complexes [40], [41]. HATs are responsible for the acetylation of histone H3 and H4 residues to promote chromatin modifications prior to transcription. Various studies have shown that plants are capable of expressing stress responsive genes in response to environmental cues through modulation of histone acetylation [8], [10], [29]. In case of *OsDREB1b* transcription there is an increase in the acetylation of histone H3 residues within promoter and upstream region. Histone H3K9 acetylation was found to be increased throughout the 800 bp region, whereas H3K14 and K27 acetylation is more biased towards core promoter and upstream region respectively (Figure 3). Previous studies on mouse embryonic stem cells suggest that enrichment of active histone modifications marks such as H3K9ac, H3K14ac, H3K27ac and H3K4me3 together with enrichment of Pol II around or at TSS is correlated with increase in gene expression [42]. The acetylation of promoter region of *OsDREB1b* locus is accompanied with enhancement of Pol II occupancy during cold stress (Figure 4A). There is no change in the H3 occupancy in these regions negating the possibility of nucleosome eviction (Figure 3A and B). In case of Arabidopsis drought responsive gene, it has been reported that there is region specific acetylation of H3K9, K14, K23 and K27 residues during transcription activation and these marks were associated with change in nucleosome occupancy at the promoter and coding region [8]. The differential acetylation of *OsDREB1b* locus thus seems to be a pre-requisite for the chromatin remodeling to facilitate cold induced expression of the gene. Our results show that the transcript level of *OsDREB1b* return to basal level in the plants which were subjected to normal growth temperature after stress treatment (Figure 7A). The repression of *OsDREB1b* transcript level after returning to normal condition is associated with reduction of histone acetylation and RNA Pol II occupancy (Figure 7B). Thus it can be concluded that the change in chromatin conformations facilitated by histone acetylation during *OsDREB1b* transcription are not independent events but are correlated processes.

This study also reveals a global reduction of H3K14 acetylation during cold stress. It has been shown that enrichment of H3K14ac and H3K9ac along with H3K27ac and H3K4me3 is associated with the active promoter sites, bivalent promoters and active enhancers [42] indicating that these histone marks are good candidates for transcription activation. Moreover, for housekeeping genes having high CpG island at the promoter region, acetylation of H3K9 and 14 residue act as a physical barrier to protect DNA methylation and methylation of H3K9 and hence enable constitutive expression of housekeeping. Recent report has shown K14 acetylation can act as docking platform for other acetyltransferase to propagate acetylation of important residues in a given loci when needed for activation under specific stimuli [43]. Now, when plants are challenged with environmental stress there is requirement for repression of certain genes (may be housekeeping or other constitutive genes) for which deacetylation of H3K14 is needed. This repression of gene expression can slow down the overall metabolism of the plants, enabling conservation of energy for overcoming the temporary challenge after stress. Although there is no direct proof for this hypothesis however a genome-wide change in H3K14 profile under cold stress can lead to more promising information about the role of H3K14 acetylation.

In this study we have observed dynamic changes in the acetylation marks of histone H3 to promote chromatin remodelling at the promoter and upstream region of *OsDREB1b* gene in response to cold stress. Our result is in consonance with previous work done on maize DREB1 during cold stress where it has been shown that hyperacetylation of ICE1 binding sites at the DREB1 upstream region leads to change in chromatin conformation [44]. Our work provides first evidence of locus specific nucleosome map and how modifications at the nucleosome level affect chromatin landscape at the *OsDREB1b* locus during cold stress response. Such studies are important because changes of chromatin structure at the promoter regions will give insight about the transcription initiation regulation of stress responsive genes. Many questions still remained to be answered like changes in DNA methylation; role of histone variants during active transcription and involvement of chromatin modifying enzymes or remodelers during stress response. A thorough study in this direction can help to understand epigenetic network involved in the transcription regulation of stress-induced genes in plants.

## Supporting Information

Figure S1 PCR based Strategy to map nucleosomes at the promoter proximal and upstream region of *OsDREB1b*. 17 days old rice seedlings were used to isolate nuclei. The nuclei were digested with micrococcal nuclease and DNA corresponding to mono- and di- nucleosome fraction was used as PCR template. A. Array of primers used to determine the position of nucleosomes between −700 to −200. B. Array of primers used to determine the position of nucleosomes between −200 to +400. The region which gives amplicon of comparable intensity from mononucleosomal DNA and genomic DNA is considered to have a positioned nucleosome as marked by asterisk (*).
(TIF)

Figure S2 Gel documentation of the ChIP PCR products for *OsDREB1b* locus. The ChIP DNA was used to amplify for different regions of *OsDREB1b* locus from control and cold treated samples. These PCR products were separated on 2% agarose gel.
(TIF)

Figure S3 Alteration of chromatin structure at the upstream region of *OsDREB2a* during cold stress. (A and B) Relative MNase accessibility in control and cold stress treated nuclei (2 Hr and 4 Hr) was detected with PCR based method. (C and D) Relative DNase I accessibility in control and cold stress treated nuclei (2 Hr and 4 Hr) was detected with PCR based method. The amount of DNA amplified at each time point was normalised to that at time 0 and plotted against time to compare the rate of degradation. (E and F) PCR products showing the amplification of upstream and promoter proximal region of *OsDREB2a* in MNase and DNase I treated nuclei isolated from control and cold treated plants.
(TIF)

Table S1 List of primers used for this study.
(DOC)
