# Correction: Differential Acetylation of Histone H3 at the Regulatory Region of *OsDREB1b* Promoter Facilitates Chromatin Remodelling and Transcription Activation during Cold Stress

**DOI:** 10.1371/journal.pone.0105229

**Published:** 2014-08-05

**Authors:** The PLOS ONE Staff

There are errors in Figure 6 and the legend for Figure 2. Please see the corrected versions here.

**Figure 2:**
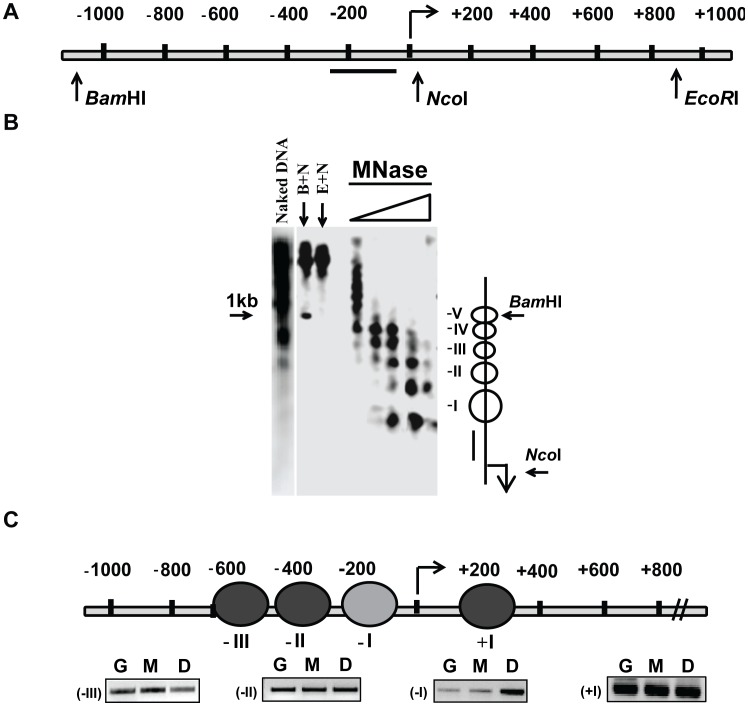
Mapping of nucleosome positions at the promoter and upstream region of *OsDREB1b* locus. (A). Schematic diagram of the *OsDREB1b* gene region and the flanking region showing the positions of the restriction enzymes that were used to map the nucleosome positions. (B). Mapping of nucleosome positions at the upstream and core promoter region of *OsDREB1b*.Chromatin isolated from 2-3 week old rice seedlings were partially digested with increasing concentration of MNase and further restriction digested with *Nco*I. The blot was probed with DNA from the core promoter region (-74 to -232) (C) Determination of the nucleosome positions at the promoter and upstream region *OsDREB1b* locus by PCR based approach. The positions of the nucleosomes are given relative to the *OsDREB1b* locus. G denotes genomic DNA, M denotes mono-nucleosomal DNA and D denotes di-nucleosomal DNA. Black shaded ovals denote a well positioned nucleosome and a grey shaded oval represents a partially positioned nucleosome.

**Figure 6:**
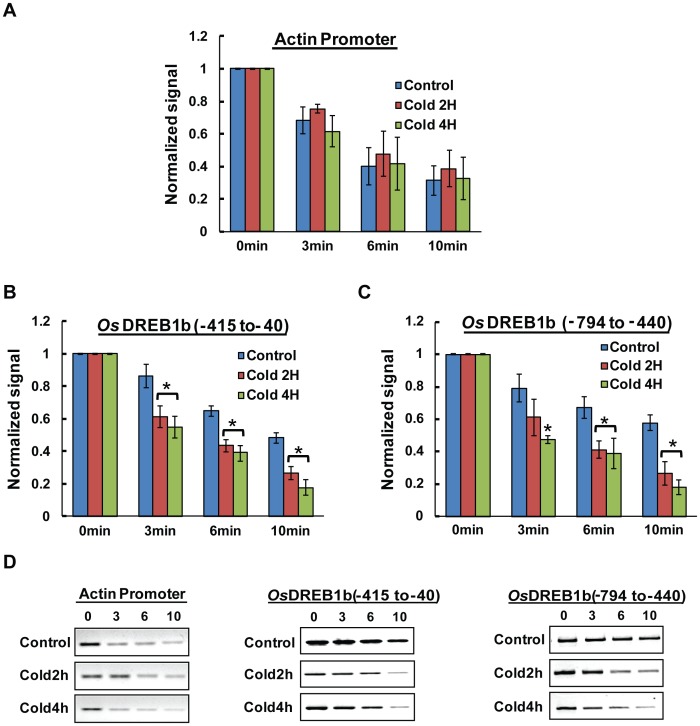
Change in DNase I accessibility at the promoter and upstream region of OsDREB1b loci. Relative DNase I accessibility in control and cold stress treated nuclei (2 Hr and 4 Hr) was detected with PCR based method. Nuclei were digested with DNase I (5 U/ml) for increasing time period (0,3,6,10 min). The isolated DNA was used for PCR reaction with primers specific for promoter and upstream region. The amount of DNA amplified at each time point was normalised to that at time 0 and plotted against time to compare the rate of degradation. The relative rate of accessibility for actin promoter (A and D) and *OsDREB1b* (B, C, D) and The data represented here is a mean of three independent experiments with standard error bars. Statistically significant values were marked with *.
