# Supplementary figures and images for: Differential Acetylation of Histone H3 at the Regulatory Region of OsDREB1b Promoter Facilitates Chromatin Remodelling and Transcription Activation during Cold Stress
Source: PLoS One. 2014 Jun 18;9(6):e100343. doi: 10.1371/journal.pone.0100343 (PMC4062490; doi:10.1371/journal.pone.0100343)

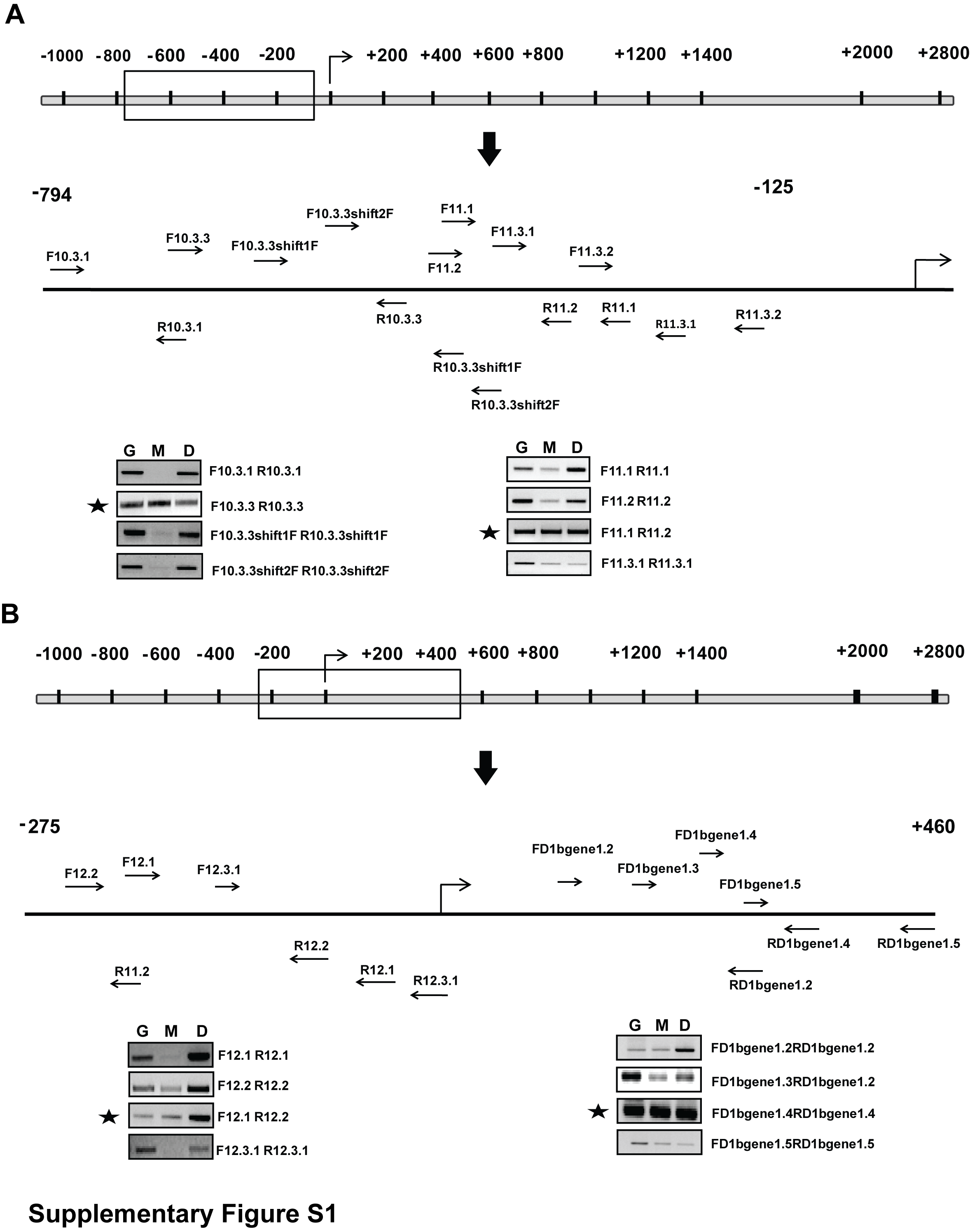

Supplement: Figure S1 — PCR based Strategy to map nucleosomes at the promoter proximal and upstream region of OsDREB1b. 17 days old rice seedlings were used to isolate nuclei. The nuclei were digested with micrococcal nuclease and DNA corresponding to mono- and di- nucleosome fraction was used as PCR template. A. Array of primers used to determine the position of nucleosomes between −700 to −200. B. Array of primers used to determine the position of nucleosomes between −200 to +400. The region which gives amplicon of comparable intensity from mononucleosomal DNA and genomic DNA is considered to have a positioned nucleosome as marked by asterisk (*). (TIF) [file pone.0100343.s001.tif]

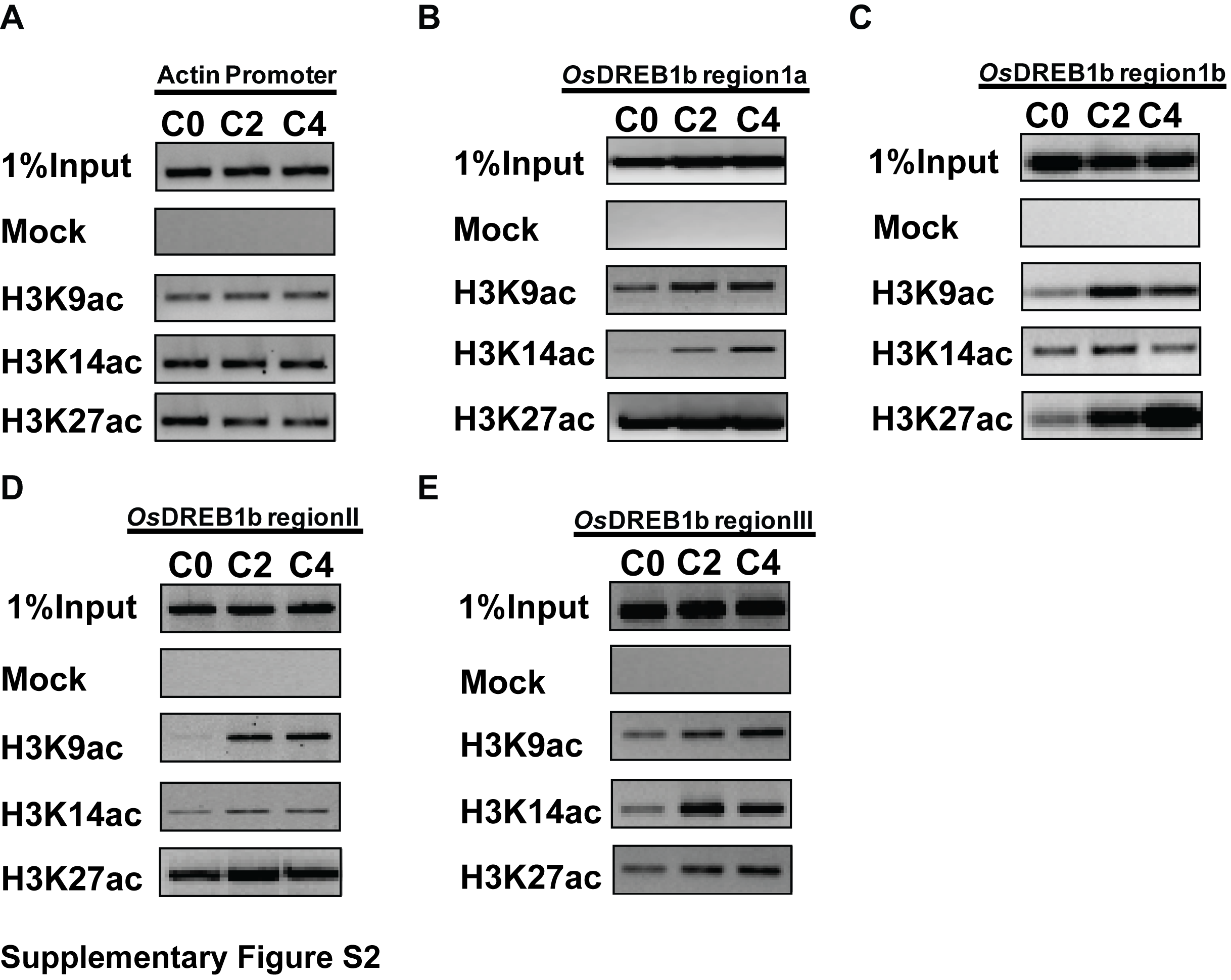

Supplement: Figure S2 — Gel documentation of the ChIP PCR products for OsDREB1b locus. The ChIP DNA was used to amplify for different regions of OsDREB1b locus from control and cold treated samples. These PCR products were separated on 2% agarose gel. (TIF) [file pone.0100343.s002.tif]

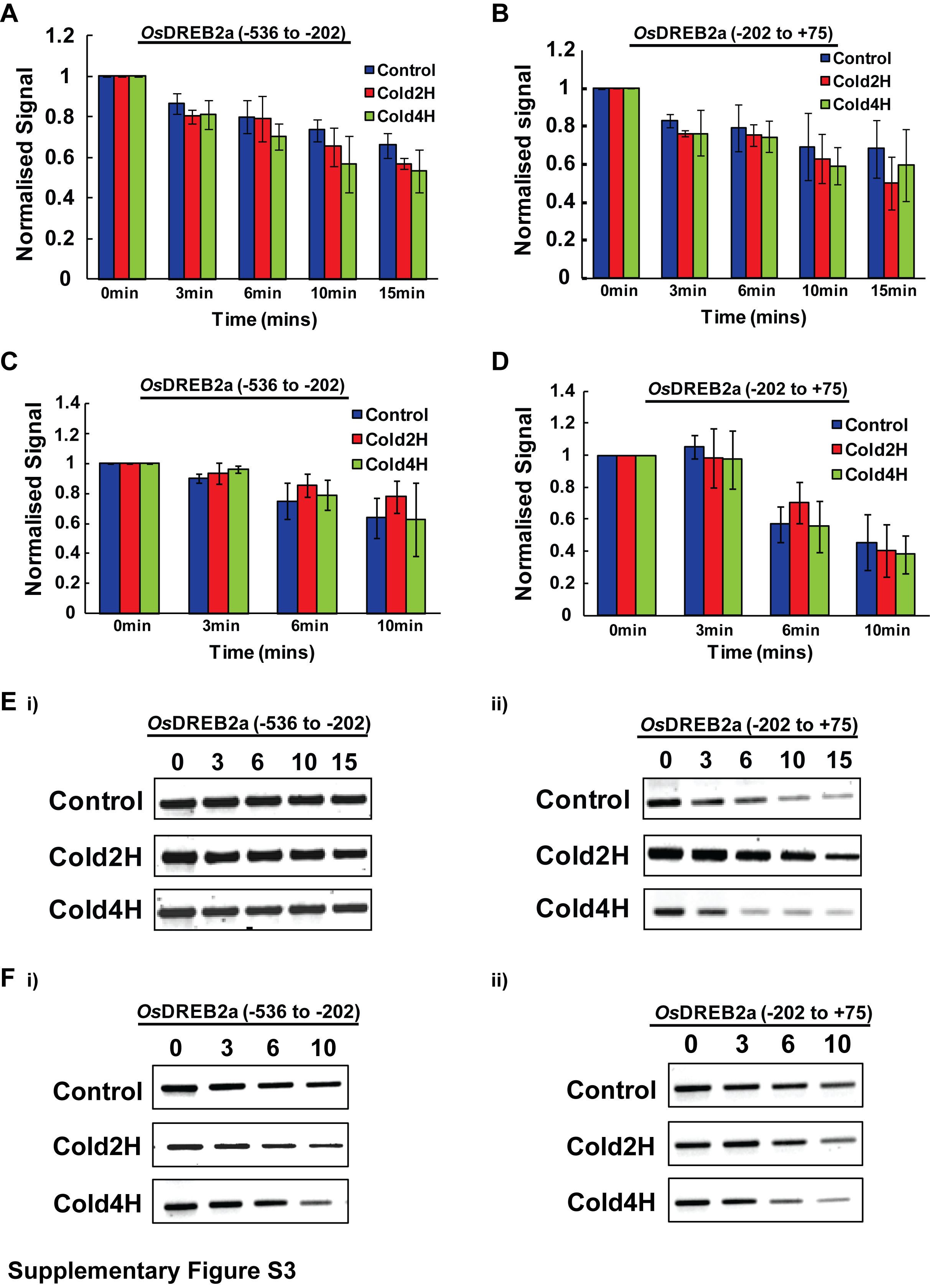

Supplement: Figure S3 — Alteration of chromatin structure at the upstream region of OsDREB2a during cold stress. (A and B) Relative MNase accessibility in control and cold stress treated nuclei (2 Hr and 4 Hr) was detected with PCR based method. (C and D) Relative DNase I accessibility in control and cold stress treated nuclei (2 Hr and 4 Hr) was detected with PCR based method. The amount of DNA amplified at each time point was normalised to that at time 0 and plotted against time to compare the rate of degradation. (E and F) PCR products showing the amplification of upstream and promoter proximal region of OsDREB2a in MNase and DNase I treated nuclei isolated from control and cold treated plants. (TIF) [file pone.0100343.s003.tif]
