# Supplementary material for: Differential Acetylation of Histone H3 at the Regulatory Region of OsDREB1b Promoter Facilitates Chromatin Remodelling and Transcription Activation during Cold Stress
Source: PLoS One. 2014 Jun 18;9(6):e100343. doi: 10.1371/journal.pone.0100343 (PMC4062490; doi:10.1371/journal.pone.0100343)
Supplement: Table S1 — List of primers used for this study. (DOC) [file pone.0100343.s004.doc]

| **Primer name** | **Location relative**  **to gene** | **Region** | **Sequence** | |
| --- | --- | --- | --- | --- |
| **Primers for Northern blot probe preparation** | | | | |
| FDREB1b | DREB1b, coding/3’UTR | +658 to +674 | 5’ CTC TGG AGC TAC TAA TC 3’ | |
| RDREB1b | DREB1b, 3’UTR | +843 to +827 | 5’ GGA ATC ACA AAA GGA GG 3’ | |
| FDREB2a | DREB2a, 3’ UTR | +3411 to +3428 | 5’ CAGGAGCGGAAGAATATGGA 3’ | |
| RDREB2a | DREB2a, 3’ UTR | +3600 to +3584 | 5’ GACCCGCAGCATGACTACTA 3’ | |
| Factin1 | OsJNBa0005K07.1, coding | +959 to +978 | 5’ CTCCAGGCATCCACGAGACG 3’ | |
| Ractin1 | OsJNBa0005K07.1, coding | +1076 to +1059 | 5’ GCGATGCCAGGGAACATG 3’ | |
| **Primers for nucleosome mapping , ChIP and nuclease accessibility** | | | | |
| F10.3.1 | DREB1b, upstream | -794 to -777 | | 5’ AGGCAAACTTAGGCTGAG 3’ |
| R10.3.1 | DREB1b, upstream | -593 to -610 | | 5’ GCACGCTTCCCAAACTAT 3’ |
| F10.3.3 | DREB1b, upstream | -610 to -593 | | 5’ ATAGTTTGGGAAGCGTGC 3’ |
| R10.3.3 | DREB1b, upstream | -440 to -457 | | 5’ CTCCAAGGTGAGACTGCA 3’ |
| F10.3.3shift1F | DREB1b, upstream | -552 to -535 | | 5’ GCGAACGCAGCCTTAGAG 3’ |
| R10.3.3shift1F | DREB1b, upstream | -380 to -398 | | 5’ GTAGTGCCGTGGTAAGGTG 3’ |
| F10.3.3shift2F | DREB1b, upstream | -522 to -501 | | 5’ CGACACTCATGGCATCTTTGAC 3’ |
| R10.3.3shift2F | DREB1b, upstream | -321 to -338 | | 5’ TCGGTTGACAGGGCTCTC 3’ |
| F11.1 | DREB1b, upstream | -415 to -396 | | 5’ GCCAAACCATACACCTCAC 3’ |
| R11.1 | DREB1b, upstream | -215 to -232 | | 5’ CTACTCCAGCTTGCAGGC 3’ |
| F11.2 | DREB1b, upstream | -457 to -440 | | 5’ TGCAGTCTCACCTTGGAG 3’ |
| R11.2 | DREB1b, upstream | -258 to -275 | | 5’ CTGTTGGGTTTTTGGGGC 3’ |
| F11.3.1 | DREB1b, upstream | -378 to -361 | | 5’ CCCATGAACCCCATCTAG 3’ |
| R11.3.1 | DREB1b, upstream | -154 to -170 | | 5’ GGCAGCTCACACTCTCG 3’ |
| F12.1 | DREB1b, upstream | -232 to -215 | | 5’ GCCTGCAAGCTGGAGTAG 3’ |
| R12.1 | DREB1b, upstream | -40 to -58 | | 5’ GACTTGGAGTTGGAGATGG 3’ |
| F12.2 | DREB1b, upstream | -275 to -258 | | 5’ GCCCCAAAAACCCAACAG 3’ |
| R12.2 | DREB1b, upstream | -74 to -91 | | 5’ AAGTGGTGGTCGTTACTC 3’ |
| F12.3.1 | DREB1b, upstream | -170 to -154 | | 5’ CGAGAGTGTGAGCTGCC 3’ |
| R12.3.1 | DREB1b, upstream | +15 to -4 | | 5’ GGATGACTCTCTCTGGTTC 3’ |
| FD1bgene1.2 | DREB1b, coding | +37 to +55 | | 5’ TACAGGACGGTGTGGTCGG 3’ |
| RD1Bgene1.2 | DREB1b, coding | +221 to +205 | | 5’ GCGAACGTGCCGAGCCA 3’ |
| FD1bgene1.3 | DREB1b, coding | +84 to +102 | | 5’ CAAGTTCAGGGAGACGAGG 3’ |
| FD1bgene1.4 | DREB1b, coding | +157 to +174 | | 5’ AGGTGGGTGTGCGAGGTG 3’ |
| RD1bgene1.4 | DREB1b, coding | +307 to +289 | | 5’ CGGAGTCGGCGAAGTTGA 3’ |
| FD1bgene1.5 | DREB1b, coding | +289 to +307 | | 5’ TCAACTTCGCCGACTCCG 3’ |
| RD1bgene1.5 | DREB1b, coding | +464 to +447 | | 5’ TCTTCTTCTTCGTCGCCA 3’ |
| F3.1Ds | DREB1b, downstream | +1192 to +1209 | | 5’ GCAAAAGTCCTCCTGAAC 3’ |
| R3.2Ds | DREB1b, downstream | +1357 to +1340 | | 5’ GGACAGAGCAATGGTGCA 3’ |
| F3.3.4Ds | DREB1b, downstream | +1409 to +1427 | | 5’ AACCTGAAGCAGACTCCAG 3’ |
| R3.3.3Ds | DREB1b, downstream | +1541 to +1522 | | 5’ CTGCAGCTAGAAGAAAGTGG 3’ |
| F2.3.3Ds | DREB1b, downstream | +1606 to +1588 | | 5’ GCCCAACGCTGCAAAAGAA 3’ |
| R2.3.2Ds | DREB1b, downstream | +1755 to +1738 | | 5’ CGTGTATGCAACTGTGTG 3’ |
| DREB2a UpF1  DREB2a UpR1  DREB2aPrF1 DREB2aPrR  F7.1  R7.1  ACTINprF  ACTINprR | DREB2a, upstream  DREB2a, upstream  DREB2a, upstream  DREB2a, 5’ UTR  DREB2a, coding  DREB2a, intron  Actin, upstream  Actin, upstream | -536 to -516  -202 to -221  -221 to -202  +1075 to +1058  +1315 to +1334  +1455 to +1437  -285 to -264  -76 to -97 | | 5’ ACCTATTATACGCGCACACAG3’  5’TTACGGCTGCTCGTGTTTAG 3’  5’TAAACACGAGCAGCCGTAAG3’  5’ CGCAGGAGCATCATGGTT3’  5’ CCA CGATGAAAGGAGGAGAT3’  5’ CTGCGCATGCATACGATG 3’  5’CATAGCAGTGAGGGTTCCATTC 3’  5’GTGCCAACGCGGAATAATTTG 3’ |

**Table: List of primers used**
